# Editorial: Interdependencies and interfaces in bone regeneration – the immune status at its core

**DOI:** 10.3389/fimmu.2024.1385796

**Published:** 2024-03-08

**Authors:** Thaqif El Khassawna, Kurt David Hankenson, Bettina Willie, Katharina Schmidt-Bleek

**Affiliations:** ^1^ Experimental Trauma Surgery, Faculty of Medicine, Justus-Liebig-University of Giessen, Giessen, Germany; ^2^ Department of Orthopaedic Surgery, University of Michigan, Ann Arbor, MI, United States; ^3^ Research Centre, Shriners Hospital for Children-Canada, Montreal, QC, Canada; ^4^ Faculty of Dental Medicine and Oral Health Sciences, McGill University, Montreal, QC, Canada; ^5^ Julius Wolff Institut for Biomechanics and Musculoskeletal Regeneration, Berlin Institute of Health at Charité – Universitätsmedizin Berlin, Berlin, Germany; ^6^ Berlin Institute of Health Centre for Regenerative Therapies, Berlin Institute of Health at Charité – Universitätsmedizin Berlin, Berlin, Germany

**Keywords:** bone regeneration, immune reaction, inflammatory phase, bone healing, immune cells, interfaces

## Incentive

Regeneration stands as the optimal outcome following an injury. However, leveraging endogenous regenerative mechanisms for therapeutic purposes, necessitates a profound understanding of the underlying processes. Researchers utilize bone as a model for regeneration, aiming to elucidate the interdependencies and interfaces within the regenerative process. Bone healing is a highly complex process that is tightly orchestrated and the immune reaction evolves as a crucial control system herein (1, 2). Distinct phases, each with unique characteristics, succeed one another, overlapping and dependent on each other, resulting in complete form and functional restoration upon successful accomplishment. However, the complexity of this intricate process exposes it to potential derailments, leading to unsatisfactory outcomes. Bone healing processes can be affected by underlying genetic, metabolic, traumatic and neoplastic conditions, all of which are interdependent with immune cell functions.

Recent findings highlight the pivotal role played by the interaction between the inflammatory response and its surrounding mechanical environment (3), metabolism (4), and revascularization (5) in the facilitating successful regenerative processes. This Research Topic provided an opportunity to compile papers exploring the regenerative process, including the healing environment beyond the scope of bone cells. Nine papers were selected from 24 submitted, comprising four original papers, four review articles, and one perspective paper. The contributions of 55 authors from around the world, including Belgium, Chile, China, Germany, New Zealand, Sweden, the United Kingdom, and the United States, are included in this Research Topic.

## Research

The articles in this Research Topic focus on a current research trend: the utilization of computational capabilities. To aid histological analysis, in silico models are being created. E. Borgiani et al. introduced COMBINI, an in silico method that allows for simulation of the early inflammatory reaction during bone healing at tissue, cell and molecular levels. The model’s output has been verified against experimental ex vivo immunofluorescent images. This innovative tool holds significant potential for exploring the mechano-biological interdependencies in the process of regenerative bone healing. Haffner-Luntzer et al. focused on altered metabolism and neuro-endocrine regulation during bone formation, emphasizing the importance of the early inflammatory response for a successful healing. They examined the impact of a concurrent brain and bone injury, particularly investigating mast cells and their involvement in osteoclastogenesis. Yang et al. utilised a bibliometric analytical approach to provide an overview of the research field concerning the interdependence of macrophages and osteoarthritis over the past 30 years. Meanwhile, Wang et al. emphasised the altered inflammatory pathways during the aging process. While inflammatory pathways are still active with progressive aging (and indeed may be overactive), signals that promote bone formation decrease. Wang et al. undertake an expression analysis harnessing several online tools and were able to thus identify a total of nine potential drugs to prevent age-related bone loss.

The original research articles emphasize the significance of the initial inflammatory healing stage, highlighting the availability of new analytical tools, due to recent advancements in computing technology. Additionally, the articles shed light on the interdependence between inflammation and biomechanics, inflammation and metabolic and endocrine signalling, inflammation and age-related bone loss and inflammation and degenerative diseases such as osteoarthritis.

## Review

Reviews in this Research Topic underscore the interdependence of the immune response in musculoskeletal conditions, further highlighting the close link between the immune system and bone homeostasis, along with the pivotal role of the immune system in pathological musculoskeletal conditions. Capobianco et al., provide an overview of approaches studying inflammatory cells in fracture healing, thereby summarizing the current knowledge of the immune-stromal crosstalk including identifying gaps that still need investigating. Zheng et al. reviewed osteoimmunology focusing on chronic inflammation and detail the pathophysiological mechanism of osteonecrosis. Altered osteoimmune functions, e.g. due to glucocorticoids or alcohol, affect bone metabolic homeostasis causing osteonecrosis. The authors propose new treatment ideas based on this literature review. Albrektsson et al. investigated the impact of osteoimmunomodulation by endosseous implants. In this context, the implant triggers a foreign body response that affects osseointegration, which can either enable or derail ingrowth, leading to peri-implant bone loss.

In the fourth review, Ren et al. introduced myeloid-derived suppressor cells (MDSCs), immature cells derived from myeloid that exhibit immunosuppressive functions. In chronic inflammation, these cells aim to counterbalance the overactive immune system. Displaying the versatility of the immune system, these cells can also differentiate into osteoclasts, further affecting bone metabolism. These reviews place the immune response at the centre of bone homeostasis in heathy and chronic inflammatory environments, proposing new therapeutic approaches to prevent bone loss in specific patient situations.

## Perspective

In the context of the research theme, a perspective article proposed a speculative hypothesis, suggesting that cell-free DNA and its activation of the innate immune response might substantially contribute to postoperative bone loss following alveolar bone grafting (Huang et al.). While cell-free DNA has been studied in the context of periodontitis, the authors speculate on its broader role in bone loss by activating the innate immune response, triggering NF-kB activation, and increased TNFa (tumor necrosis factor alpha) expression. TNFa serves as a marker cytokine for pro-inflammatory processes. Cell-free DNA includes endogenous nuclear and mitochondrial DNA, along with exogenous bacterial or viral DNA, representing a DAMP (danger-associated molecular pattern) that would be highly present in an injury situation.

## Conclusion

This Research Topic highlights the significance of the inflammatory response, particularly the initial reaction, in relation to bone formation. Furthermore, it emphasizes the interdependence and interaction of factors such as mechanics, endocrine signalling, degenerative co-morbidities, chronic inflammation, ageing, and osteoimmunology. The CRC 1444 “Directed Cellular Self‐Organisation to Advance Bone Regeneration” clarifies interdependencies and expands on the research that has been initiated within this Research Topic.

## Author contributions

TK: Writing – original draft, Writing – review & editing. KH: Writing – original draft, Writing – review & editing. BW: Writing – original draft, Writing – review & editing. KS-B: Writing – original draft, Writing – review & editing.
